# Spatial Pattern and Determinants of the First Detection Locations of Invasive Alien Species in Mainland China

**DOI:** 10.1371/journal.pone.0031734

**Published:** 2012-02-21

**Authors:** Dingcheng Huang, Runzhi Zhang, Ke Chung Kim, Andrew V. Suarez

**Affiliations:** 1 CAS Key Laboratory of Zoological Systematics and Evolution, Institute of Zoology, Chinese Academy of Sciences, Beijing, China; 2 Graduate University of Chinese Academy of Sciences, Beijing, China; 3 Department of Entomology, Pennsylvania State University, University Park, Pennsylvania, United States of America; 4 Department of Animal Biology, Department of Entomology, University of Illinois, Urbana, Illinois, United States of America; University College London, United Kingdom

## Abstract

**Background:**

The unintentional transport of species as a result of human activities has reached unprecedented rates. Once established, introduced species can be nearly impossible to eradicate. It is therefore essential to identify and monitor locations where invaders are most likely to establish new populations. Despite the obvious value of early detection, how does an agency identify areas that are most vulnerable to new invaders? Here we propose a novel approach by using the “first detection location” (FDL) of introduced species in China to quantify characteristics of areas where introduced species are first reported.

**Methodology/Principal Findings:**

We obtained FDLs for 166 species (primarily agricultural and forestry pests) that were unintentionally introduced into China prior to 2008 from literature searches. The spatial pattern and determinants of FDLs were examined at the provincial level. The spatial pattern of FDLs varied among provinces with more commerce and trade and economically developed provinces in coastal regions having more FDLs than interior provinces. For example, 74.6% of FDLs were distributed in coastal regions despite that they only cover 15.6% of the total area in China. Variables that may be indicators of “introduction pressure” (e.g. the amount of received commerce) had an overwhelming effect on the number of FDLs in each province (*R* ^2^ = 0.760).

**Conclusions/Significance:**

Our results suggest that “introduction pressure” may be one of the most important factors that determine the locations where newly-introduced species are first detected, and that open and developed provinces in China should be prioritized when developing monitoring programs that focus on locating and managing new introductions. Our study illustrates that FDL approaches can contribute to the study and management of biological invasions not only for China but also for elsewhere.

## Introduction

Despite considerable prevention efforts, new introductions of alien species continue throughout the world, particularly in areas with high levels of trade and economic development [1]–[6]. Invasive species can be difficult or nearly impossible to eradicate once they have become established in a new area. Therefore, early detection and rapid response (EDRR) is essential for preventing and containing new outbreaks [7]–[9]. Given that increasing levels of international commerce are commonly expected to accelerate the rate at which species are transported [10], [11], there is an immediate need to improve the effectiveness of EDRR strategies.

Early detection is inherently difficult as newly introduced species usually occur at low densities [7], [9], [12]. High sampling intensity increases the likelihood of successfully detecting rare species [13]. However, a trade-off exists between sampling intensity at any one location and the number of locations searched due to limited resource availability for EDRR efforts [14]. Increasing sampling intensity in areas highly vulnerable to invasion is more effective than searching a wider range with low sampling intensity [15]. Thus, effective EDRR programs that aim at locating and managing newly introduced species will depend on having accurate information on where new species are most likely to become established.

Successful EDRR efforts will also depend upon close collaboration among agencies and organizations at various levels, as new introductions often spread rapidly across jurisdictional boundaries. Thus, the establishment of a national (or even international) EDRR network system may be ideal [16], [17]. To develop such a system, however, key questions need to be addressed including: (1) where should regional EDRR stations be housed? (2) How should EDRR resources be allocated? (3) What is the geographic distribution of sites where newly introduced species are most likely to become established? The success of EDRR efforts depends upon our capacity to predict potential new introductions across regional scales.

In this study, we use the spatial pattern and abiotic determinants of “first detection locations” (FDLs) of introduced species in mainland China (hereafter termed China) to answer these key questions. We define an FDL as the location where a species of alien origin is detected and identified for the first time in a recipient country or region. FDLs of many alien pests are often reported in or near urban areas and ports of entry. For example, the FDL of *Anoplophora glabripennis* (Motschulsky) in the United States is in the urban area of New York City [18]. Non-random pattern in the distribution of FDLs can provide crucial information for focusing limited resources for identifying recently established invasive species and guiding EDRR efforts.

## Materials and Methods

### Focal organisms

As the purpose of this study is to develop a dataset of FDLs that may guide strategies for early detection of invasive species, we focus only on species that, (1) are non-native to China, (2) were unintentionally introduced into China, and (3) have been recognized as invasive in the sense that their introduction to China has been accompanied by economic loss, environmental damage and/or harm to human health [19]. Non-native species *intentionally* introduced into China were not included as the location of their establishment was chosen rather accidental. Species that have been intercepted in imported commerce but have not established in the wild were also excluded. Focusing our efforts on the three criteria above allows us to develop a framework useful for identifying areas where species of the highest concern may become established.

### Data collection

The FDLs of our focal organisms were derived from literature searches, particularly checklists of “invasive alien species” (IAS) in China. We gave priority to the primary literature for reports of the first detection and identification of an IAS, and then used other literature which documented clearly the location where a specific IAS was detected and identified for the first time in China.

Our dataset included FDLs for 166 IASs in China (Table S1). They are mostly agricultural and forestry pests across a wide range of taxa, including plants, insects, fungi, bacteria, and viruses. Thirty-two of these records did not provide detailed information on the exact location of FDL so were excluded from subsequent analyses. The remaining species were divided into three groups: plants, animals and the others (including fungi, bacteria, chromista and virus). The numbers of FDLs of each group in each provincial administrative unit (hereafter termed province) were calculated. As the numbers of FDLs of the three groups in each province were significantly correlated with their pooled data (plants: R = 0.869, *P*<0.001; animals: R = 0.866, *P*<001; others: R = 0.608, *P*<0.001), we used the pooled data to represent the combination of these three groups in the analysis outlined below.

A suite of ecological and socio-economic data by province were collected from publications [20]–[22] and grouped into five categories: (a) introduction pressure, (b) anthropogenic disturbance, (c) search and recording effort, (d) ecological/bio-geographical variability and (e) spread by unintentional introduction (Table 1). Here we used a broad categorization, as some variables could be placed into multiple categories [11]. Introduction pressure (also termed propagule pressure) was used to describe the pressure of a region to receive individuals of alien species [23].

**Table 1:** List of explanatory variables in China by province.

Category^a^	Code	Variable (Unit)^b^
DI, IP	GC	Gross domestic product per capita (1.0 thousand RMB per person)
DI, IP	GD	Gross domestic product (100 billion RMB)
DI, IP	UR	Urbanization rate (%)
DI, SE	NA	Non-agricultural population (1.0 million person)
DI, SE	PD	Population density (1000 person/km^2^)
DI, SE	PO	Population (1.0 million person)
DI, SE	UP	Urban population (1.0 million person)
EB	AN	Annual precipitation (mm)
EB	AR	Area (10 000 km^2^)
EB	AT	Mean annual temperature (°C)
EB	EN	Endemism score
EB	FC	Forest coverage (%)
EB	JA	Mean January temperature (°C)
EB	JU	Mean July temperature (°C)
EB	RH	Mean annual relative humidity (%)
IP	AP	Number of air ports of entry
IP	BF	Batch of EEIQ^c^ freight (1000 Batch)
IP	EV	Export value of commodities (100 million USD)
IP	FE	Foreign exchange earnings (1.0 million USD)
IP	IT	Number of international tourists (10 000 person-times)
IP	IV	Value of imported commodities (100 million USD)
IP	LP	Number of land ports of entry
IP	NC	Number of cities with ports of entry (individual)
IP	NP	Number of ports of entry (individual)
IP	VF	Value of EEIQ freight (1.0 million USD)
IP	WP	Number of water ports of entry
SE	ES	Expenditures for scientific research^d^ (1.0 million RMB)
SE	FS	Funds for scientific research (1.0 million RMB)
SE	SS	Staffs for scientific research (1000 Person)
SI	DF	Domestic freight traffic (1.0 million tons)
SI	DP	Domestic passenger traffic (1.0 million person-times)

a DI: Disturbance; EB: Ecological/bio-geographical variance; IP: Introduction pressure; SE: Search and recording effort; SI: Spread by unintentional introduction.

b Data of variables except EN, AP, WP, LP, NC and NP were collected from National Bureau of Statistics of China (1986–2007) China statistical yearbook. The mean values of these variables were used for data analysis. Endemism score (EN) means the total values of endemism of species including plants, mammals and birds in each province, collected from McBeath G.A & Leng T.K. (2006) Governance of Biodiversity Conservation in China and Taiwan. Information about AP, WP, LP, NC and NP was collected from China Association of Port-of-Entry (2003) Practical Manual of Ports of Entry in China.

c EEIQ: Entry-Exit Inspection and Quarantine.

d Scientific research refers to state-owned research and development institutions above county level in the field of natural sciences and technology.

### Spatial pattern of FDLs

**Figure 1:**
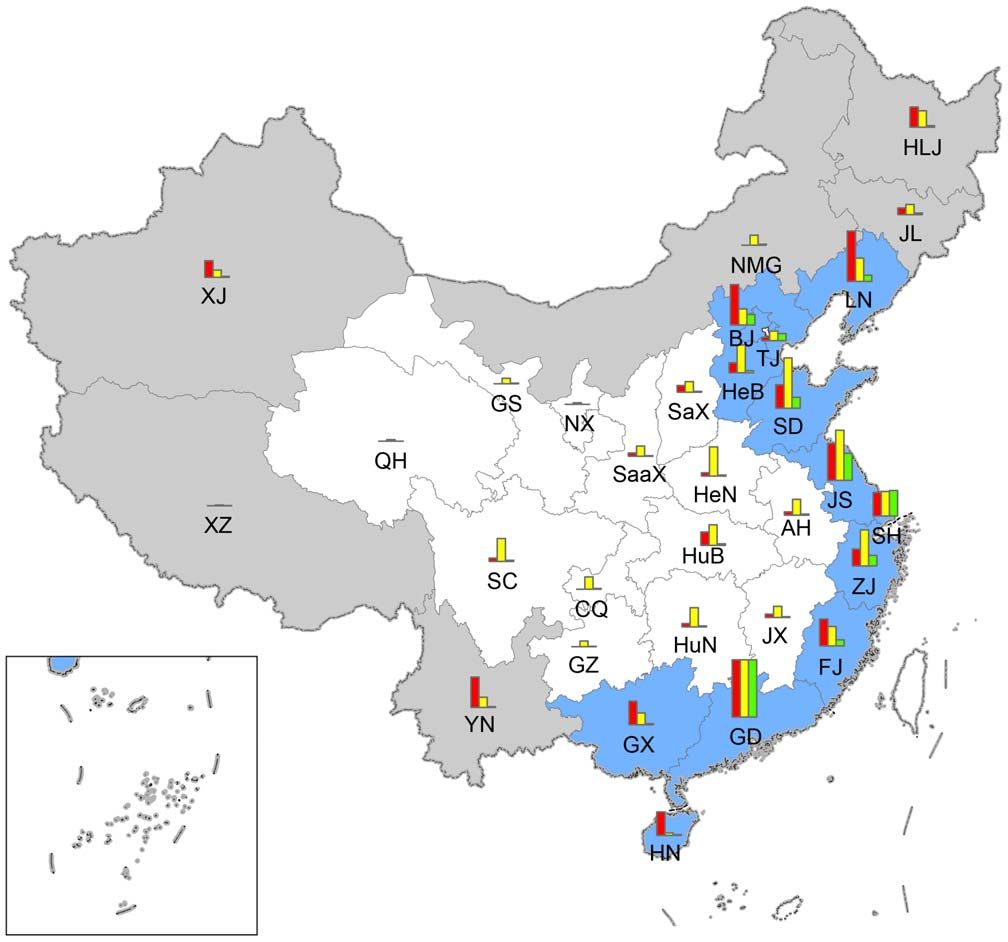
Distribution of first detection locations of invasive alien species in mainland China. Provincial administrative units in mainland China were separated into three groups according to their geographic position: coastal region in blue ( = provinces with sea coasts except Beijing), border region in grey ( = provinces continuous to other countries) and midland region in white ( = provinces without sea coasts or borders on other countries). Bars in red are the number of first detection locations in each province. Bars in yellow and green (for the average GDP and import value of commodities from 1986 to 2007, respectively) are standardized with same height in Guangdong province which has the highest GDP and the highest number of first detection locations. AH, BJ, CQ, FJ, GS, GD, GX, GZ, HeB, HeN, HLJ, HN, HuB, HuN, JL, JS, JX, NMG, NX, QH, SD, SaX, SaaX, SC, SH, TJ, XJ, XZ, YN and ZJ are provinces codes, standing for Anhui, Beijing, Chongqing, Fujian, Gansu, Guangdong, Guangxi, Guizhou, Hebei, Henan, Heilongjiang, Hainan, Hubei, Hunan, Jilin, Jiangsu, Jiangxi, Inner Mongolia, Ningxia, Qinghai, Shandong, Shanxi, Shaanxi, Sichuan, Shanghai, Tianjin, Xinjiang, Tibet, Yunnan and Zhejiang, respectively.

To examine the spatial pattern of FDLs at provincial level, we divided provinces in China into three groups according to their geographic positions: coastal regions ( = provinces with sea coasts except Beijing), border regions ( = inland provinces that border other countries) and midland regions (provinces without sea coasts or borders adjacent to other countries) (Fig. 1). Kruskal-Wallis test was used to compare the differences in the number of FDLs in each province among the three regions, as not all these data could be transformed to assume a normal distribution.

### Determinants of FDL distribution

Spearman rank correlation was used to identify potential relationships between variables used in this study. As many explanatory variables were highly interrelated (Table S2), and relationships between these variables may be strongly nonlinear and involve high-order interactions, we built regression trees [24]–[26] to explain the spatial pattern of FDLs. The trees were built by repeatedly splitting the response variable (i.e. the number of FDLs in each province) using binary recursive partitioning in CART v. 6.0.

To find the best tree, 50 sets of 10-fold cross-validation were run, on the basis of the minimum cost tree rule, which minimizes the cross-validated relative error, and on the one-SE rule, which minimizes cross-validated relative error within one SE of the minimum, respectively [24]. The most frequent (most likely) single tree was chosen for description. Ten-fold cross-validation was used to estimate the cross-validated relative error and resubstituion relative error of this tree. Total variance of response variable explained by the best tree was calculated as *R* ^2^ = 1−resubstitution relative error. The quality of each split was corresponding to proportion of the total sum of squares explained by the tree at each node.

## Results

### Spatial pattern

The distribution of FDLs varied among provinces with more commerce and trade and economically developed provinces in coast regions having more FDLs than provinces in border and midland regions (Fig. 1). For example, the number of FDLs was highest in Guangdong, a coastal province (17), whereas its neighbor Hunan Province, a midland province had only one. By comparison, Hunan is 0.2 times larger than Guangdong but only produces 33.4% and 1.5% of Guangdong's GDP and import value (respectively). FDLs were mostly (74.6% of the total) distributed in coastal regions which occupied only 15.6% of the total land area of China. The mean number of FDLs per province in coastal region was 8.3±1.4 (mean ± SE), 1.2 times higher than that in border region (3.7±1.5) and 8.2 times higher than that in midland region (0.9±0.3). Kruskal-Wallis test showed their difference was significant (*χ* ^2^ = 16.565, *P*<0.000). These results indicated that IASs were more likely detected first in coastal region compared to border or midland regions.

### Determinants

The number of FDLs in each province was significantly related to variables that may be indicators of introduction pressure (e.g. the number of water ports of entry, the number of international tourists) and variables that reflect human disturbance on recipient regions (e.g. population density, gross domestic product per capita per capita) (Table S2).

**Figure 2:**
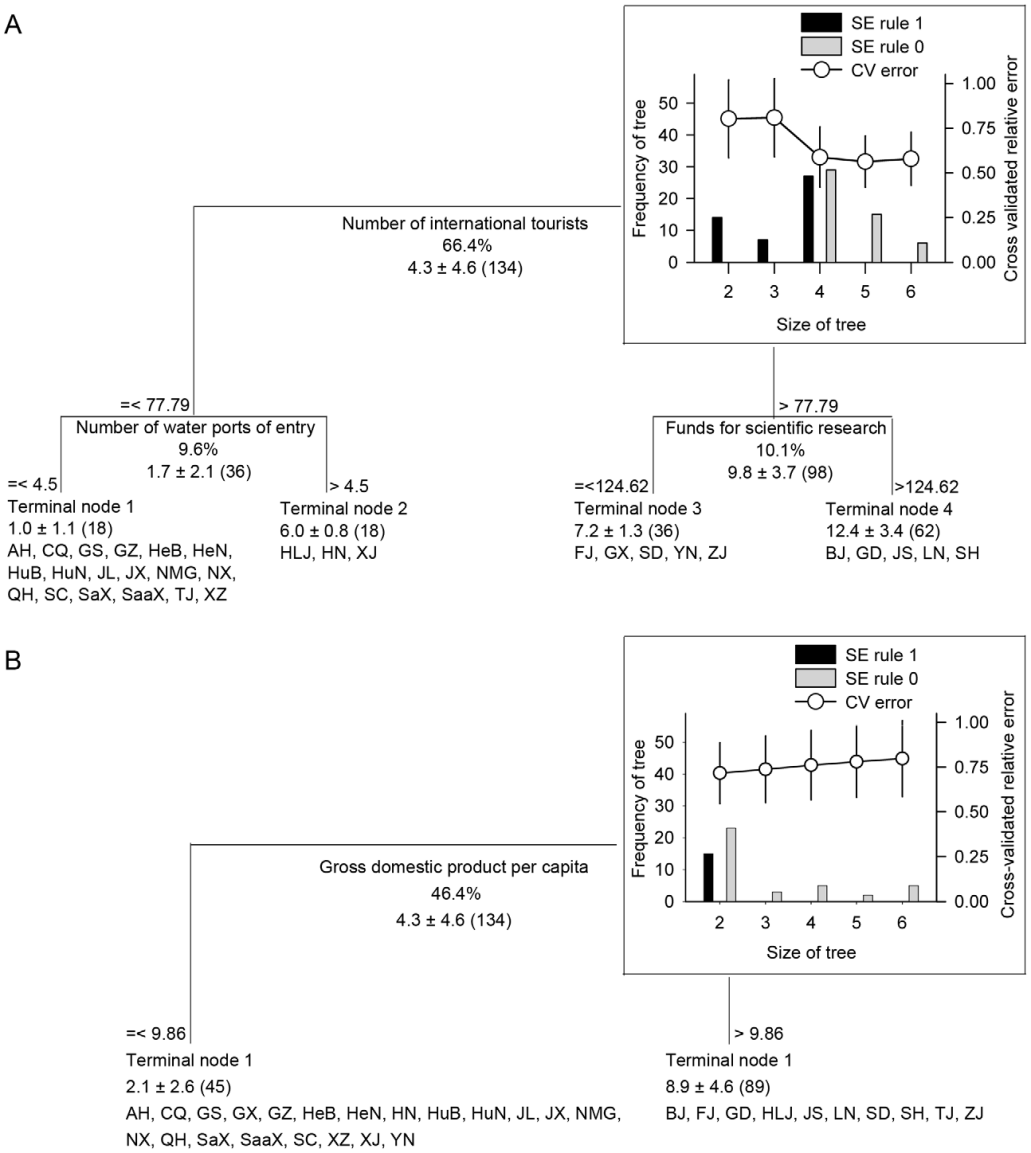
Regression tree analysis for the determinants of first detection location of invasive alien species. A: using all explanatory variables; B: using explanatory variables except those classified into “IP” category (Table 1). Each node of the tree is described by the splitting variable, its splitting criteria, percentage of variance the splitter explains, mean ± standard deviation for the number of first detection locations of invasive alien species, and the number of sample (i.e. species) at that node in brackets. (*Inset*) Cross-validation processes for selection of the best regression trees. Line shows a single representative 10-fold cross-validation of the most frequent (modal) best trees with standard error (SE) estimates of each tree size. Bar charts are the numbers of the optimal trees of each size (frequency of tree) selected from a series of 50 cross-validations based on the minimum cost tree, which minimizes the cross-validated relative error (white, SE rule 0), and 50 cross-validations based on the one-SE rule (gray, SE rule 1), which minimizes the cross-validated relative error within one SE of the minimum. The most frequent trees have four terminal nodes. See the legend of Fig. 1 for province codes.

Regression trees further support this pattern. When all explanatory variables were included, the cross-validated relative error of the best tree was 0.563, indicating that this model had predictive capacity of the FDL distribution. The tree explained 86.1% of variance, of which 76.0% was attributed to two variables that represent introduction pressure, and 10.1% to funds for scientific research that reflects search and recording effort (Fig. 2 A). As variables of other categories (e.g., gross domestic product per capita) were important and strong competitors of the first splitter (Table S3), we removed all variables of the “IP” category (Table 1) to explore whether the effects of other factors were masked ((or “squeezed out”)) by variables representing introduction pressure. The cross-validated relative error of the resulting best tree was 0.717. It had only two terminal nodes, split by gross domestic product per capita that explained 46.4% of variance (Fig. 2 B). No other predictive trees were constructed (i.e. cross-validated relative error >1.0), when we removed all variables of the “IP” and “DI, IP” categories.

## Discussion

### Species number and available data

Compiling a list of IASs for a given recipient region is a helpful approach to understand patterns of invasion [27]. Because we focused only on the species that were unintentionally introduced into and have evidently done damage in China, our species list certainly represents only a fraction of all alien species in China. Having a list of all alien species and their FDLs would be indispensable, but is presently not feasible to obtain. This reflects a knowledge gap that needs to be filled and also the difficulty in collecting accurate FDLs for species once they have been established for a long period of time. As is the case in many countries, there is currently no central repository for records of invasive species in China.

While our species list is incomplete, it is likely representative of the larger species pool of IASs that were unintentionally introduced into China. The number of IASs in China is currently unknown although a checklist published in 2004 listed 283 species (including intentionally and unintentionally introduced IASs, and those that naturally dispersed into China), a likely underestimate [28], [29]. For example, previous checklists [30], [31] do not include invaders discovered in recent years, such as *Solenopsis invicta* Buren and *Phenacoccus solenopsis* Tinsley. Nascent lists may have a larger number of IASs [32]–[34]. However, they include species native to China, such as *Coptotermes formosanus* Shiraki that become alien and invasive out of their native range within China, and contain those that have been intercepted but not yet established in China, such as *Anthonomus grandis* (Boheman). Future efforts will benefit from drawing upon both a larger data set of species and also from including more regions either nationally or internationally.

### FDL spatial distribution

Our results suggest that IASs unintentionally introduced into China were more frequently first detected in coastal provinces. This pattern is similar to those revealed in previous studies [1], [35], [36], showing that the numbers of IASs in coastal provinces are higher than those in inland provinces. However, previous results find the distribution pattern of introduced species to be correlated significantly with domestic traffic and climate, and strongly associated with economic development but weakly with international transport [1]. In contrast, our results strongly implicated international transport and other forms of “propagule pressure” (Fig. 2 A, Table S2).

It is important to note that many our focal IASs, such as *Liriomyza sativae* Blanchard and *Lissorhoptrus oryzophilus* Kuschel, have invaded midland provinces. However, few were first detected in this region [30], [31], [37]. One possible explanation to these contrasting patterns could be due to patterns of spread; IASs entering midland regions may be detected after spreading from coastal provinces rather than the reverse. Support for this may come from the fact that variables representing spread by domestic transport were weakly correlated with the number of FDLs in each province (Table S2) and explained little of the variance in the number of FDLs (not appeared in the optimal trees, Fig. 2 A).

In our view, a more reasonable explanation could be that most IASs found in midland provinces spread from frontier provinces, and most IASs initially entering coastal provinces were discovered prior to their detections in midland provinces, as coastal regions had much higher introduction pressure (with 91.0% of the total import value, 78.6% of the total international tourists in China), which accounted for most of the variance that was much higher than those explained by other factors (Fig. 2 A). This concurs with previous studies suggesting that a booming economy sparks and accelerates biological invasions in China [1], [38].

### Factors that affect FDL distribution

Theoretically, FDLs may be determined by many factors including international transport, domestic traffic, biological traits of species, native biodiversity, climatic suitability, and search and recording effort. In comparison to these latent factors, our results appeared somewhat unexpected. These variables were weakly related with our response variable (Table S2), and did not appear in the best regression trees (Fig. 2 A, B). This does not mean that those factors have no roles in the FDL distribution. They may affect the FDL distribution at larger or lower scales.

Our study showed an overwhelming effect of variables representing introduction pressure on the number of FDLs in each province (Table S2, Fig. 2 A), indicating that introduction pressure is one of the most important determinants on the FDL distribution. This result is in agreement with a commonly accepted paradigm that international transport plays an important role in the spread of IASs [39]–[41].

Significant associations of the number of FDLs in each province with population density (Table S2) and the presence of funds for scientific research in the regression tree (Fig. 2 A) suggest that search and recording effort may be an important determinant of where introduced species are first detected. This has been demonstrated in many IASs such as *S. invicta* [42] and *Bursaphelenchus xylophilus* (Steiner and Buhre) [43], which were first discovered in areas where people were already aware of their potential presence and hazards before first detection.

High degree of disturbance can increase the vulnerability of recipient ecosystems [44]. This favorable factor could lead to an increase of species relative abundance, which is an important determinant of the discovery of introduced species [45]. Our study revealed that variables such as gross domestic product per capita, urbanization rate that may be indicators of disturbance [11] were significantly related with the number of FDLs. Also, gross domestic product per capita was the third strongest competitor of the first splitter of the best regression tree (Fig. 2 A, Table S3) and explained a considerable fraction of the variance in the number of FDLs (Fig. 2 B). These results suggest that disturbance may be another important determinant of the FDL distribution.

### Implications for managing new introductions

As the international transport between China and other regions continues to grow, the number of new introductions will increase. Many species listed in the Global Invasive Species Database are posing potential threats to China [46]. The patterns revealed in our study can be useful in developing programs that aim at locating and managing new introductions. For example, when designing a national EDRR network system, regional stations should be prioritized in coastal and developed provinces. It is particularly worth directing more EDRR resources to Bohai Rim, Yangtze River Delta and Pearl River Delta, because these areas are widely open, highly developed and disturbed in China.

The differences between present distribution pattern and first detection pattern show that domestic transport may have accelerated the spread of IASs in China [38]. This highlights the importance of post-border inspection and interception that aim to reduce the unintentional introductions between provinces.

### Expanding this approach to other scales

Here we provided a case study to illustrate how FDL approach can contribute to the study of biological invasions. This approach can be expanded at both larger and smaller scales. At global scale, more variables can be investigated and their relative importance assessed. For example, across continents the relative importance of commerce can be compared to abiotic variables. Biological invasions are conceptually considered depending on propagule pressure and abiotic suitability [47]. Their relative importance in the determination of establishment success may be estimated by this approach. At national scale, we can compare the similarity and differences of the distribution patterns of FDLs in various countries to find common patterns and rules. At landscape scale, we can quantify the habitat type and ecosystem type of the FDLs and better understand their relationships with the biological traits of IASs.

## Supporting Information

Table S1 **List of invasive alien species and their first detection locations in mainland China.**
(DOC)

Table S2 **Full Spearman rank correlation matrix of all variables used in this study.**
(DOC)

Table S3 **Relative importance of explanatory variables and root node competitors in order of improvement for the regression tree in** **Fig. 2 A** **.**
(DOC)
