# Supplementary material for: Spatial Pattern and Determinants of the First Detection Locations of Invasive Alien Species in Mainland China
Source: PLoS One. 2012 Feb 21;7(2):e31734. doi: 10.1371/journal.pone.0031734 (PMC3283667; doi:10.1371/journal.pone.0031734)
Supplement: Table S1 — List of invasive alien species and their first detection locations in mainland China. (DOC) [file pone.0031734.s001.doc]

**Table S1.** List of invasive alien species and their first detection locations (FDLs) in mainland China. Species tabulated here were unintentionally introduced into China.

| Scientific name | FDL | Ref |
| --- | --- | --- |
| Animalia |  |  |
| Arachnida |  |  |
| Prostigmata |  |  |
| Tetranychidae |  |  |
| *Tetranychus urticae* Koch, 1836 | Beijing | [1] |
| Bivalvia |  |  |
| Veneroida |  |  |
| Dreissenidae |  |  |
| *Mytilopsis sallei* (Recluz, 1849) | Fujian | [2] |
| Gastropoda |  |  |
| Neotaenioglossa |  |  |
| Calyptraeidae |  |  |
| *Crepidula onyx* G. B. Sowerby I, 1824 | Hong Kong | [3] |
| Stylommatophora |  |  |
| Achatinidae |  |  |
| *Achatina fulica* (Ferussac, 1821) | Fujian | [4] |
| Insecta |  |  |
| Blattodea |  |  |
| Blattellidae |  |  |
| *Blattella germanica* (L., 1767) | Northeast China | [5] |
| Blattidae |  |  |
| *Periplaneta americana* (L., 1758) | Taiwan | [6] |
| *Periplaneta australasiae* (F., 1775) | Liaoning | [7] |
| Coleoptera |  |  |
| Bostrychidae |  |  |
| *Heterobostrychus aequalis* (Waterhouse, 1884) | Guangdong | [3] |
| Brentidae |  |  |
| *Cylas formicarius* Olivier, 1807 | Zhejiang | [8] |
| Bruchidae |  |  |
| *Acanthoscelides macrophthalmus* (Schaeffer, 1907) | Hainan | [9] |
| *Acanthoscelides obtectus* (Say, 1859) | Liaoning | [10] |
| *Bruchus pisorum* (L., 1758) | Hebei | [3] |
| *Callosobruchus maculatus* (F., 1775) | Zhejiang | [2] |
| *Zabrotes subfasciatus* (Boheman, 1833) | Guangdong | [11] |
| Chrysomelidae |  |  |
| *Brontispa longissima* (Gestro, 1885) | Guangdong | [12] |
| *Ophraella communa* LeSage, 1986 | Jiangsu | [13] |
| Curculionidae |  |  |
| *Axionicus insignis* Pascoe, 1869 | Guangxi | [14] |
| *Dendroctonus valens* LeConte, 1859 | Shanxi | [3] |
| *Lissorhoptrus oryzophilus* Kuschel, 1952 | Hebei | [3] |
| *Rhabdoscelus lineaticollis* (Heller) | Guangdong | [15] |
| *Rhynchophorus ferrugineus* Herbst, 1795 | Guangdong | [6] |
| *Sitophilus granarius* (L., 1758) | Shichuan | [16] |
| Hispidae |  |  |
| *Octodonta nipae* (Maulik, 1921) | Hainan | [17] |
| Diptera |  |  |
| Agromyzidae |  |  |
| *Liriomyza bryoniae* (Kaltenbach, 1858) | Shanghai | [18] |
| *Liriomyza huidobrensis* (Blanchard, 1926) | Yunnan | [6] |
| *Liriomyza sativae* Blanchard, 1938 | Hainan | [6] |
| *Liriomyza trifolii* Burgess, 1880 | Guangdong | [19] |
| Cecidomyiidae |  |  |
| *Mayetiola destructor* Say, 1817 | Xinjiang | [20] |
| *Obolodiplosis robiniae* Haldeman, 1847 | Liaoning | [21] |
| Tephritidae |  |  |
| *Bactrocera dorsalis* Hendel, 1912 | Hainan | [22] |
| *Carpomya vesuviana* Costa, 1854 | Xinjiang | [23] |
| Hemiptera |  |  |
| Aleyrodidae |  |  |
| *Aleurodicus dispersus* Russell, 1965 | Hainan | [24] |
| *Bemisia argentifolii* Bellows and Perring, 1994 | Shanghai | [25] |
| *Bemisia tabaci* (Gennadius, 1889) | South China | [3] |
| *Bemisia tabaci* (Gennadius, 1889) Q biotype | Yunnan | [26] |
| *Trialeurodes vaporariorum* (Westwood, 1856) | Beijing | [5] |
| Aphididae |  |  |
| *Eriosoma lanigerum* (Hausmann, 1802) | Shandong | [27] |
| Diaspididae |  |  |
| *Hemiberlesia pitysophila* Takagi, 1969 | Guangdong | [3] |
| Margarodidae |  |  |
| *Matsucoccus matsumurae* (Kuwana, 1905) | Liaoning | [28] |
| *Icerya aegyptiaca* (Douglas, 1890) | Taiwan | [29] |
| Phylloxeridae |  |  |
| *Daktulosphaira vitifoliae* (Fitch, 1855) | Shandong | [3] |
| Pseudococcidae |  |  |
| *Oracella acuta* (Lobdell, 1930) | Guangdong | [3] |
| *Phenacoccus solenopsis* Tinsley, 1898 | Guangdong | [30] |
| *Dysmicoccus neobrevipes* Beardsley, 1959 | Hainan | [31] |
| Tingidae |  |  |
| *Corythucha ciliata* (Say, 1832) | Hubei | [32] |
| Hymenoptera |  |  |
| Eulophidae |  |  |
| *Leptocybe invasa* Fisher and LaSalle, 2004 | Guangxi | [33] |
| *Quadrastichus erythrinae* Kim, 2004 | Guangdong | [34] |
| Formicidae |  |  |
| *Solenopsis invicta* Buren, 1972 | Guangdong | [35] |
| Tenthredinidae |  |  |
| *Nematus melanaspis* Hartig, 1840 | Shanxi | [36] |
| Isoptera |  |  |
| *Incisitermes minor* (Hagen, 1858) | Zhejiang | [6] |
| Lepidoptera |  |  |
| Arctiidae |  |  |
| *Hyphantria cunea* (Drury, 1773) | Liaoning | [37] |
| Gelechiidae |  |  |
| *Phthorimaea operculella* Zeller, 1873 | Guangxi | [38] |
| Tineidae |  |  |
| *Opogona sacchari* Bojer, 1856 | Beijing | [3] |
| Tortricidae |  |  |
| *Cydia pomonella* L., 1758 | Xinjiang | [39] |
| Thysanoptera |  |  |
| Thripidae |  |  |
| *Frankliniella occidentalis* (Pergande, 1895) | Beijing | [40] |
| Malacostraca |  |  |
| Decapoda |  |  |
| Cambaridae |  |  |
| *Procambarus clarkii* (Girard, 1852) | Jiangsu | [41] |
| Isopoda |  |  |
| Sphaeromatidae |  |  |
| *Sphaeroma walkeri* Stebbing, 1905 | Hong Kong | [3] |
| Mammalia |  |  |
| Rodentia |  |  |
| Muridae |  |  |
| *Rattus norvegicus norvegicus* (Berkenhout, 1769) | South China | [3] |
| Maxillopoda |  |  |
| Sessilia |  |  |
| Balanidae |  |  |
| *Balanus amphitrite* Darwin, 1854 | Shandong | [3] |
| *Balanus eburneus* Gould, 1841 | Shandong | [3] |
| *Balanus improvisus* Darwin, 1854 | Shandong | [3] |
| Secernentea |  |  |
| Aphelenchida |  |  |
| Aphelenchidae |  |  |
| *Bursaphelenchus xylophilus* (Steiner and Buhrer, 1934) | Jiangsu | [42] |
| Aphelenchoididae |  |  |
| *Aphelenchoides ritzemabosi* (Schwartz, 1911) | Jiangsu | [3] |
| Tylenchida |  |  |
| Anguinidae |  |  |
| *Ditylenchus destructor* Thorne, 1945 |  |  |
| Hoplolaimidae |  |  |
| *Radopholus similis* (Cobb, 1893) | Fujian | [43] |
| Bacteria |  |  |
| Actinobacteria |  |  |
| Actinomycetales |  |  |
| Microbacteriaceae |  |  |
| *Clavibacter michiganensis* subsp. *michiganensis* (Smith, 1910) | Beijing | [3] |
| *Clavibacter michiganensis* subsp. *sepedonicus* (Spieckermann and Kotthoff, 1914) | Heilongjiang | [44] |
| Betaproteobacteria |  |  |
| Burkholderiales |  |  |
| Burkholderiaceae |  |  |
| *Ralstonia solanacearum* (Smith, 1896) Yabuuchi et al., 1996 | Guangxi | [3] |
| Gammaproteobacteria |  |  |
| Pseudomonadales |  |  |
| Pseudomonadaceae |  |  |
| *Pseudomonas savastanoi* (Janse, 1982) Gardan et al., 1992 | Guangxi | [3] |
| Xanthomonadales |  |  |
| Xanthomonadaceae |  |  |
| *Xanthomonas campestris* pv. *vesicatoria* (Doidge, 1920) Dye, 1978 | Hunan | [45] |
| *Xanthomonas oryzae* pv. *oryzae* (ex Ishiyama, 1922) Swings et al., 1990 | Jiangsu | [5] |
| *Xanthomonas oryzae* pv. *oryzicola* (Fang et al., 1957) Swings et al., 1990 | Guangdong | [3] |
| Chromista |  |  |
| Oomycetes |  |  |
| Peronosporales |  |  |
| Peronosporaceae |  |  |
| *Peronosclerospora sacchari* (T. Miyake) Shirai and Hara, 1927 | Taiwan | [46] |
| *Phytophthora sojae* Kaufm. and Gerd., 1958 | Beijing | [47] |
| *Plasmopara halstedii* (Farl.) Berl. and De Toni, 1888 | Heilongjiang | [48] |
| Fungi |  |  |
| Chytridiomycetes |  |  |
| Chytridiales |  |  |
| Synchytriaceae |  |  |
| *Synchytrium endobioticum* (Schilb.) Percival, 1909 | Yunnan | [3] |
| Dothideomycetes |  |  |
| Botryosphaeriales |  |  |
| Botryosphaeriaceae |  |  |
| *Botryosphaeria laricina* (Sawada) Y.Z. Shang | Heilongjiang | [3] |
| Capnodiales |  |  |
| Davidiellaceae |  |  |
| *Cladosporium cucumerinum* Ellis and Arthur 1889 | Henan | [49] |
| Mycosphaerellaceae |  |  |
| *Mycosphaerella pini* Rostr., 1957 | Heilongjiang | [3] |
| *Mycosphaerella dearnessii* M.E. Barr, 1972 | Fujian | [50] |
| Pleosporales |  |  |
| Leptosphaeriaceae |  |  |
| *Leptosphaeria lindquistii* Frezzi, 1968 | Xinjiang | [45] |
| Pleosporaceae |  |  |
| *Alternariaster helianthi* (Hansf.) E.G. Simmons, 2007 | Jilin | [51] |
| Venturiaceae |  |  |
| *Spilocaea oleaginea* (Castagne) S. Hughes, 1953 | Yunnan | [3] |
| Leotiomycetes |  |  |
| Erysiphales |  |  |
| Erysiphaceae |  |  |
| *Podosphaera aphanis* (Wallr.) U. Braun and S. Takam., 2000 | Liaoning | [45] |
| Helotiales |  |  |
| Hyaloscyphaceae |  |  |
| *Lachnellula willkommii* (Hartig) Dennis, 1962 | Heilongjiang | [3] |
| Pucciniomycetes |  |  |
| Pucciniales |  |  |
| Cronartiaceae |  |  |
| *Cronartium ribicola* J.C. Fisch., 1872 | Liaoning | [3] |
| Pucciniaceae |  |  |
| *Puccinia horiana* Henn., 1901 | Shanghai | [52] |
| Sordariomycetes |  |  |
| (unrank) |  |  |
| Plectosphaerellaceae |  |  |
| *Verticillium albo-atrum* Reinke and Berthold, 1879 | Xinjiang | [53] |
| *Verticillium dahliae* Kleb., 1913 | Jiangsu | [54] |
| Diapthorales |  |  |
| Gnomoniaceae |  |  |
| *Cryptodiaporthe populea* (Sacc.) Butin ex Butin, 1958 | Jiangsu | [3] |
| Hypocreales |  |  |
| Nectriaceae |  |  |
| *Calonectria kyotensis* Terash., 1968 | Guangxi | [3] |
| *Fusarium oxysporum dianthi* W.C. Snyder and H.N. Hansen, 1940 | Shanghai | [3] |
| *Fusarium oxysporum* f. sp. *cubense* | Guangxi | [45] |
| *Fusarium oxysporum vasinfectum* W.C. Snyder and H.N. Hansen, 1940 | Jiangsu | [54] |
| Microascales |  |  |
| Ceratocystidaceae |  |  |
| *Ceratocystis fimbriata* Ellis and Halst., 1890 | Liaoning | [45] |
| Plantae |  |  |
| Liliopsida |  |  |
| Poales |  |  |
| Poaceae |  |  |
| *Avena fatua* L., 1753 | Fujian | [6] |
| *Cenchrus spinifex* Cavanilles, 1799 | Liaoning | [55] |
| *Lolium temulentum* L., 1753 | Heilongjiang | [56] |
| *Panicum repens* L., 1762 | Hong Kong | [6] |
| *Paspalum conjugatum* Bergius, 1772 | Hong Kong | [6] |
| *Sorghum halepense* (L.) Pers., 1805 | Guangdong | [57] |
| Magnoliopsida |  |  |
| Apiales |  |  |
| Apiaceae |  |  |
| *Cyclospermum leptophyllum* (Pers.) Sprague ex Britt. and Wilson, 1925 | Hong Kong | [3] |
| *Daucus carota* L., 1753 | Macao | [5] |
| *Eryngium foetidum* L., 1753 | Yunnan | [6] |
| Asterales |  |  |
| Asteraceae |  |  |
| *Acanthospermum australe* (Loefl.) Kuntze, 1891 | Yunnan | [6] |
| *Ambrosia artemisiifolia* L., 1753 | Zhejiang | [6] |
| *Ambrosia trifida* L., 1753 | Liaoning | [3] |
| *Bidens frondosa* L., 1753 | Zhejiang | [3] |
| *Bidens pilosa* L., 1753 | Hong Kong | [6] |
| *Chromolaena odorata* (L.) King and H.E. Robinson, 1970 | Yunnan | [3] |
| *Flaveria bidentis* (L.) Kuntze, 1898 | Tianjin | [58] |
| *Galinsoga parviflora* Cav., 1795 | Yunnan | [6] |
| *Gymnostyles anthemifolia* Juss., 1817 | Hong Kong | [3] |
| *Iva xanthifolia* Nutt | Liaoning | [59] |
| *Parthenium hysterophorus* L., 1753 | Yunnan | [3] |
| *Praxelis clematidea* (Griseb.) R. M. King et H. Rob | Guangdong | [6] |
| *Senecio vulgaris* L., 1753 | Northeast China | [3] |
| *Symphyotrichum subulatum* (Michx.) Nesom, 1995 | Hubei | [3] |
| *Synedrella nodiflora* (L.) Gaertn., 1791 | Hong Kong | [3] |
| *Tridax procumbens* L., 1753 | Guangdong | [6] |
| *Xanthium spinosum* (L.) | Beijing | [3] |
| *Xanthium strumarium* var. *canadense* (P. Mill.) Torr. and Gray | Beijing | [60] |
| *Xanthium strumarium* var. *glabratum* (DC.) Cronq. | Beijing | [61] |
| Campanulales |  |  |
| Campanulaceae |  |  |
| *Triodanis perfoliata* (L.) Nieuwl. | Fujian | [56] |
| *Triodanis perfoliata* var. *biflora* (Ruiz and Pavón) Bradley | Anhui | [56] |
| Capparales |  |  |
| Brassicaceae |  |  |
| *Coronopus didymus* (L., 1753) | Jiangsu | [3] |
| *Lepidium virginicum* L., 1753 | Hubei | [3] |
| Caryophyllales |  |  |
| Amaranthaceae |  |  |
| *Alternanthera paronychioides* A. St.-Hil., 1833 | Guangdong | [62] |
| *Alternanthera pungens* Kunth, 1818 | Fujian | [3] |
| *Amaranthus albus* L., 1759 | Northeast China | [56] |
| *Amaranthus blitoides* S. Watson, 1877 | Liaoning | [56] |
| *Amaranthus palmeri* S. Watson, 1877 | Beijing | [63] |
| *Amaranthus polygonoides* L., 1759 | Shandong | [6] |
| *Amaranthus spinosus* L., 1753 | Hong Kong | [3] |
| *Amaranthus viridis* L., 1763 | Taiwan | [3] |
| Caryophyllaceae |  |  |
| *Agrostemma githago* L., 1753 | Northeast China | [3] |
| *Stellaria apetala* Ucria ex Roem, 1796 | Jiangsu | [64] |
| Chenopodiaceae |  |  |
| *Chenopodium ambrosioides* L., 1753 | Taiwan | [3] |
| *Chenopodium simplex* (Torrey) Rafinesque, 1832 | Hebei | [6] |
| Geraniales |  |  |
| Geraniaceae |  |  |
| *Geranium carolinianum* L., 1753 | East China | [6] |
| Lamiales |  |  |
| Lamiaceae |  |  |
| *Hyptis rhomboidea* M. Martens and Galeotti, 1844 | Hainan | [6] |
| *Hyptis suaveolens* (L.) Poit., 1806 | Taiwan | [6] |
| Verbenaceae |  |  |
| *Stachytarpheta jamaicensis* (L.) Vahl, 1805 | Hong Kong | [6] |
| Malpighiales |  |  |
| Euphorbiaceae |  |  |
| *Euphorbia dentata* Michx., 1803 | Beijing | [6] |
| *Euphorbia hirta* L., 1753 | Macao | [6] |
| *Euphorbia maculata* L., 1753 | Shanghai | [6] |
| Malvales |  |  |
| Malvaceae |  |  |
| *Malvastrum coromandelianum* (L.) Garcke, 1857 | Guangdong | [3] |
| Sterculiaceae |  |  |
| *Waltheria indica* L., 1753 | Hong Kong | [6] |
| Onagraceae |  |  |
| *Oenothera laciniata* Hill., 1767 | Shanghai | [65] |
| Piperales |  |  |
| Piperaceae |  |  |
| *Peperomia pellucida* (L.) Kunth, 1815 | Hong Kong | [5] |
| Plantaginales |  |  |
| Plantaginaceae |  |  |
| *Plantago aristata* Michx., 1803 | Shandong | [56] |
| *Plantago virginica* L., 1753 | Jiangxi | [6] |
| Scrophulariales |  |  |
| Scrophulariaceae |  |  |
| *Veronica hederifolia* L. | Jiangsu | [66] |
| *Veronica persica* Poiret, 1808 | Hubei | [3] |
| Solanales |  |  |
| Convolvulaceae |  |  |
| *Ipomoea cairica* (L.) Sweet, 1826 | Hong Kong | [6] |
| Solanaceae |  |  |
| *Physalis pubescens* L., 1753 |  |  |
| *Solanum capsicoides* Allioni, 1773 | Hong Kong | [6] |
| *Solanum erianthum* D. Don, 1825 | Fujian | [6] |
| *Solanum rostratum* Dunal | Liaoning | [67] |
| *Solanum torvum* Swartz, 1788 | Macao | [6] |
| Urticales |  |  |
| Urticaceae |  |  |
| *Pilea microphylla* (L.) Liebm., 1851 | Taiwan | [6] |
| Violales |  |  |
| Cucurbitaceae |  |  |
| *Sicyos angulatus* L. | Liaoning | [68] |
| Passifloraceae |  |  |
| *Passiflora foetida* L., 1753 | Hong Kong | [6] |
| Virus |  |  |
| Group II (ssDNA) |  |  |
| Geminiviridae |  |  |
| Tomato yellow leaf curl virus Cohen and Harpaz | Shanghai | [69] |
| Group IV ((+)ssRNA) |  |  |
| (Unrank) |  |  |
| Cucumber green mottle mosaic virus Ainsworth | Liaoning | [70] |
| Bromoviridae |  |  |
| Prunus necrotic ringspot virus | Shaanxi | [71] |
| Comoviridae |  |  |
| Tobacco ring spot virus (Chandrasekar and Johnson) | Jilin | [72] |
| Flexiviridae |  |  |
| Poplar mosaic virus (Cagelli and Lefèvre) | Beijing | [73] |

**References**

1. Zhang N, Dong H, Chen J, Deng X (1983) Effects of prey species and relative humidity on the development, survival and fecundity of three species of redacious mites. J Plant Prot 10: 103–108.
2. Ding J, Mack RN, Lu P, Ren M, Huang H (2008) China's booming economy is sparking and accelerating biological invasions. BioScience 58: 317–324.
3. Xu H, Qiang S (2004) Checklist of invasive alien species in China. Beijing: China Environmental Science Press. 432 p.
4. Chen D, Zhang W (2004) Alien species *Achatina fulica*. Bull Biol 39: 15–16.
5. Xu Z, Chen W, Cai G (2008) Identification and control of invasive alien species in Hangzhou District. Hangzhou: Zhejiang University Press. 189 p.
6. Li Z, Xie Y (2002) Invasive Alien Species in China. Beijing: China Forestry Publishing House. 211 p.
7. Zhang S, Zhao Y (1996) Geographical Distribution of Agricultural and Forestry Insects in China. Beijing: China Agricultural Press. 400 p.
8. Zhong P (1994) The occurrence of *Cylas formicarius* in Zhejiang and its quarantine approaches. Plant Quar 8: 304–305.
9. Qin X, Zhang S, Zhang Z, Chen Z, Tang J, et al. (2007) A new invasive insect pest *Acanthoscelides macrophthalmus* (Coleoptera: Bruchidae). Chin J Trop Crops 28: 101–103.
10. Wang J, Wang X, Lv F, Gao P (2006) Risk analysis of *Acathoscelides obtectus* in China. Chin Plant Prot 26: 8–11.
11. Zhu W, Deng Y (1991) A preliminary study on the biology of *Zabrotes subfasciatus* (Bruchidae, Coleoptera). J Southwest Agric Univ 13: 243–246.
12. Zhang Z, Cheng D, Jiang D, Xu H (2004) Spread, damage and control methods of *Brontispa longissima*. Entomol Knowl 41: 522–526.
13. Meng L, Li B (2005) Advances on biology and host specificity of the newly introduced beetle, *Ophraella communa* Lesage (Coleoptera: Chrysomelidae), attacking *Ambrosia artemisiifollia* (Compositae) in continent of China. Chin J Biol Control 21: 65–69.
14. Li H, Han H, Zhang R, Xue D (2005) List of invasive alien insects in mainland China. In: Qiao G, Chen H, Xiao H, editors. Research Advance in Entomology. Beijing. pp. 10–17.
15. Wang G, Chen J, Han R (2005) Advances in the research on the biology and control of coconut weevil *Rhabdwscelus lineaticollis* (Heller). Nat Enemies Insects 27: 127–133.
16. Li L, Lu L, Liao S (1961) Discovery of *Sitophilus grannaria* (L.) in Szechuan. Acta Entomol Sin 15: 19.
17. Sun J, Yu P, Zhang Y, Wang X (2003) A new invasive coconut pest in Hainan Province. Entomol Knowl 40: 286–287.
18. Wu J, Zeng L, Liang G, Zhang W (1996) Studies on *Liriomyza bryoniae* (Diptera: Agromyzidae) and its natural enemies: a review. Nat Enemies Insects 18: 43–46.
19. Wang X, Huang D, Li H, Xue D, Zhang R, et al. (2006) Invasion and identification of *Liriomyza trifolii* and its potential distribution areas in China. Chin Bull Entomol 43: 540–545.
20. Liang Y (1980) Discovery of *Mayetiola destructor* in northwestern China. Plant Quar 6: 2.
21. Yang Z, Qiao X, Bu W, Yao Y, Xiao Y, et al. (2006) First discovery of an important invasive insect pest, *Obolodiplosis robiniae* (Diptera: Cecidomyiidae) in China. Acta Entomol Sin 49: 1050–1053.
22. Zia Y (1937) Study on the Trypetidae of fruit-flies of China. Sinenia 8: 103–217.
23. Adili S, He S, Tian C, Luo Y, Yu F, et al. (2008) The ocurrence and pupae distribution pattern of *Carpomya vesuviana* in Turfan area. Plant quar 22: 295–297.
24. Yu G, Zhang G, Peng Z, Liu K, Fu Y (2007) The spiralling whitefly, *Aleurodicus dispersus*, invaded Hainan Island of China. Chin Bull Entomol 44: 428–431.
25. Liu S (2007) Invasion biology and sustainable management of *Bemisia tabaci*. In: Li D, Wu C, Wu Y, Meng X, editors. Proceedings of the 8th National Congress and Annual Meeting of the Entomological Society of China. Beijing: China Agricultural science and technology press. pp. 629.
26. Chu D, Zhang Y, Brown JK, Cong B, Xu B, et al. (2006) The introduction of the exotic Q biotype of *Bemisia tabaci* from the mediterranean region into China on ornamental crops. Fla Entomol 89: 168–174.
27. Sun L, Tan X, Zhou H, Gu S, Guo J, et al. (2008) Investigation methods for population size of *Eriosoma lanigerum* in the orchard. Chin Bull Entomol 45: 818–822.
28. Zhao S, Chang G, Dang Z (1990) The occurrence and control strategy of *Matsucoccus matsumurae* in China. For Sci Technol 19: 1–3.
29. Zhao G, Zhou W, Wu Y (1994) Nonnative plant pathogens and insect pests in Taiwan. Taiwan Agric Res 11: 25–27.
30. Wu S, Zhang R (2009) A new invasive pest, *Phenacoccus solenopsis*, threatening seriously to cotton production. Chin Bull Entomol 46: 159–162.
31. Zhang X, Chen Z, Zhong Y, Wu H (2008) Elementary study on the life habits of *Dysmicoccus neobrevipes* (Beardsley). Entomol J East Chin 17: 22–25.
32. Li C, Xia W, Wang F (2007) First records of *coryithucha ciliata* in China (Hemiptera, Tingidae). Acta Zootaxonomica Sin 32: 944–946.
33. Wu Y, Jiang X, Li D, Luo J, Zhou G, et al. (2009) *Leptocybe invasa*, a new invasive forest pest making galls on twigs and leaves of *Eucalyptus* Trees in China (Hymenoptera: Eulophidae). Sci Silvae Sin 45: 161–163.
34. Yang W, Yu D, Jiao Y, Chen Z, Yang X (2005) First report of a new invasive pest, *Quadrastichus erythrinae* Kim in China. Plant Prot 31: 93.
35. Zeng L, Lu Y, He X, Zhang W, Liang G (2005) Identification of red imported fire ant *Solenopsis invicta* to invade mainland China and infestation in Wuchuan, Guangdong. Chin Bull Entomol 42: 144–148.
36. Qin Q, Miao Z, Tong Y (1994) A preliminary study on the biology of *Nematus melanaspis* Hartig. For Pest Dis 13: 22–23.
37. Chen Z, Zhang S, Li Y, Wang J, Ai D (1980) Fall webworm (*Hyphantria cunea* Drury): an invasive pest newly introduced into China. Plant Prot 6: 25.
38. Li X, Jin X, Li Z (2005) The present status and developing tendency in *Phthorimaea operculella* research. J Qinghai Norm Univ (Nat Sci Ed) 27: 67–70.
39. Chang H (1953) Taxonomic notes on the codling moth, *Carpocapsa pomonella* L. in Sinkiang. Acta Entomol Sin 7: 467–472.
40. Zhang Y, Wu Q, Xu B, Zhu G (2003) The occurrence and damage of *Frankliniella occidentalis* (Thysanoptera: Thripidae) in Beijing. Plant Prot 23: 58–59.
41. Wan F, Guo J, Zhang F (2009) Research on Biological Invasions in China. Beijing: Science Press. 302 p.
42. Sun Y (1982) The pine wilt nematode (*Bursaphelenchus xylophilus*) detected at Sun Yet-sen's mausoleum in Nanjing. J Jiangsu For Sci Technol 9: 47.
43. Li Y, Li F, Luo H, Ke H (2006) On the management of invasive alien species, banana burrowing nematode (*Radopholus similis*). Plant Protection 32: 119–121.
44. Lv W (1998) *Corynebacterium sepedonicum*. J Potato 12: 125–126.
45. Zhang G, Fu W, Liu K (2008) Major Invasive Alien Species in Agricultural. Beijing: Science Press. 409 p.
46. Zhou W, Cai J (1993) Introduction of three sugarcane diseases in Taiwan. Plant Quar 7: 271–272.
47. Su Y, Shen C (1993) The discovery and biological characteristics studies of *Phytophthora megasperm* f. sp. *glycinea* on soybean in China. Acta Phytopathol Sin 23: 341–347.
48. Zhou Z, Yan J (1989) The investigation and identification of *Plasmopara halstedii* in China. Plant Quar 3: 108–110.
49. Li M, Yi Q (1989) The occurence of *Cladosporium cucumerinum* in China. J Changjiang Vegetables 6: 26–27.
50. Li C, Zhu X, Han Z, Zhang J, Shen B, et al. (1986) Investigation on brown-spot needle blight of pines in China. J Nanjing For Univ (Nat Sci Ed) 29: 11–18.
51. Yu L, Zhang L, Li C, Guan C (1995) *Alternaria helianthi* on sunflower was identified and compared its forms with its closed species. J Jilin Agric Univ 18: 22–24.
52. Wang S, Wang H, Lei Z, Dai S (2008) Review of Chrysanthemum white rust. North Hortic 32: 67–70.
53. Ma D, Qin X, Wang P, Ma Y, Jia Y, et al. (2006) The isolated outbreak and eradication of *Verticillium albo-atrum* Reinke & Berthold in Xinjiang area. Plant Quar 20: 394.
54. Hong X, Xu H, Li H, Xie L (2003) Alien invasive insects and pathogens in Jiangsu Province: current status, influence and control. J Nanjing Agric Univ 26: 116–123.
55. Wang W, Han Z (2005) The damage and distribution of invasive alien species, *Cenchrus panciflorus* in Liaoning area. Pratacultural Sci 22: 63–64.
56. Liu L, Zhu T, Chen W, Wu Z, Lu S (2002) Flora reipublicae popularis Sinicae (Tomus 9) Angiospermae, Monocotyledoneae: Gramineae (Poaceae) (2). Beijing: Science Press. 450 p
57. Zhang J (1991) The difference between *Sorghum halepense* and its related species. Plant Quar 5: 12–16.
58. Gao X, Tang T, Liang Y, Zheng T, Sang W, et al. (2004) An alert regarding biological invasion by a new exotic plant, *Flaveria bidentis* and strategies for its control. Biodivers Sci 12: 274–279.
59. Guan G (1983) A new invasive alien weed in China: *Iva xanthifolia* Nutt. Plant Quar 5: 44–49.
60. Jindian C, Hu B (2007) An invasive alien weed *Xanthium italicum* Moretti. Weed Sci: 58–59.
61. Che J, Sun G (1992) Two new weeds discovered in Beijing: *Xanthium strumarium* var. *glabratum* and *X. italicum*. Chin Plant Prot 12: 39–40.
62. Wu T (1994) A Checklist of Flowering Plants of Islands and Reefs of Hainan and Guangdong Provinces. Beijing: Science Press.
63. Li Z (2003) *Amaranthus palmeri* S. Watson, a newly naturalized species in China. Chin Bull Bot 20: 734–735.
64. Gu D, Xu B (1989) A study on the populations of *Stelloria media* and *S. apetala* in Nanjing area. Guihaia 9: 265–270.
65. Jiang M, Ding B, Cao J, Liu Q (2004) Alien weed: *Oenothern laciniata*. Plant Quar 18: 285–287.
66. Chen S, Liu S (1986) Keys to tracheophyte species in Jiangsu, China. Nanjing: Jiangsu Scientific and Technological Press. pp. 498–499.
67. Lin Y, Tan D (2007) The potential and exotic invasive plant: *Solanum rostratum*. Acta Phytotaxon Sin 45: 675–685.
68. Wang Q, Li Y, Chen C (2005) *Sicyos*, a naturalized genus of Cucurbitaceae in mainland China. Acta Bot Boreali-Occidentalia Sinica 25: 1227–1229.
69. Wu J, Dai F, Zhou X (2006) First report of Tomato yellow leaf curl virus in China. Plant Dis 90: 1359.
70. Chen J, Li M (2007) A new invasive species: Cucumber green mottle mosaic virus. Plant Quar 21: 94–96.
71. Wei N, Wu Y (1989) Identification of flower virus disease (II). J Yunnan Agric Univ 4: 302–308.
72. Chen Y, Hu W, Nan J (1990) Identification of tobacco ringspot virus on *Gladiolus gandavensis*. Acta Phytopathol Sin 20: 241–246.
73. Xiang Y (1982) An investigation on poplar mosaic virus. For Sci Technol 18: 27–30.
