# Supplementary material for: Spatial Pattern and Determinants of the First Detection Locations of Invasive Alien Species in Mainland China
Source: PLoS One. 2012 Feb 21;7(2):e31734. doi: 10.1371/journal.pone.0031734 (PMC3283667; doi:10.1371/journal.pone.0031734)
Supplement: Table S2 — Full Spearman rank correlation matrix of all variables used in this study. (DOC) [file pone.0031734.s002.doc]

**Table S2. Full Spearman rank correlation matrix of all variables used in this study. Bold indicates coefficient significance level (2-tailed), *P* < 0.05.**

| Variable ^a^ | FDL | AR | PO | PD | NA | UP | UR | EN | FC | JA | JU | AT | RH | AN | AP |
| --- | --- | --- | --- | --- | --- | --- | --- | --- | --- | --- | --- | --- | --- | --- | --- |
| AR | -0.271 |  |  |  |  |  |  |  |  |  |  |  |  |  |  |
| PO | 0.247 | 0.161 |  |  |  |  |  |  |  |  |  |  |  |  |  |
| PD | **0.436** | **-0.719** | **0.383** |  |  |  |  |  |  |  |  |  |  |  |  |
| NA | **0.427** | 0.063 | **0.716** | **0.401** |  |  |  |  |  |  |  |  |  |  |  |
| UP | **0.374** | -0.009 | **0.922** | **0.521** | **0.816** |  |  |  |  |  |  |  |  |  |  |
| UR | **0.579** | **-0.593** | 0.001 | **0.620** | **0.355** | 0.330 |  |  |  |  |  |  |  |  |  |
| EN | 0.289 | 0.330 | 0.034 | -0.213 | -0.127 | -0.027 | -0.085 |  |  |  |  |  |  |  |  |
| FC | 0.265 | 0.019 | **0.370** | 0.038 | 0.054 | **0.377** | 0.195 | **0.472** |  |  |  |  |  |  |  |
| JA | 0.201 | -0.164 | 0.333 | 0.305 | -0.098 | 0.264 | -0.020 | **0.585** | **0.543** |  |  |  |  |  |  |
| JU | 0.292 | **-0.414** | **0.440** | **0.652** | **0.355** | **0.537** | **0.402** | 0.078 | **0.429** | **0.628** |  |  |  |  |  |
| AT | 0.275 | -0.303 | **0.417** | **0.474** | 0.067 | **0.403** | 0.158 | **0.430** | **0.560** | **0.941** | **0.823** |  |  |  |  |
| RH | 0.145 | -0.199 | **0.402** | 0.341 | 0.092 | 0.380 | 0.150 | **0.433** | **0.535** | **0.754** | **0.617** | **0.791** |  |  |  |
| AN | **0.395** | -0.267 | **0.421** | **0.484** | 0.156 | **0.480** | 0.323 | **0.477** | **0.672** | **0.855** | **0.756** | **0.894** | **0.817** |  |  |
| AP | **0.534** | 0.028 | **0.442** | 0.187 | 0.348 | 0.444 | 0.211 | 0.375 | **0.513** | 0.296 | 0.328 | 0.348 | 0.265 | **0.387** |  |
| WP | **0.733** | -0.015 | **0.412** | 0.225 | **0.516** | **0.478** | **0.371** | 0.313 | **0.358** | 0.187 | 0.326 | 0.274 | 0.251 | **0.379** | **0.772** |
| LP | 0.099 | **0.364** | -0.050 | **-0.392** | -0.019 | -0.006 | 0.055 | 0.323 | 0.240 | -0.183 | **-0.447** | -0.278 | -0.245 | -0.125 | 0.272 |
| NP | **0.614** | 0.158 | 0.345 | 0.049 | **0.446** | **0.411** | 0.279 | 0.374 | 0.353 | 0.080 | 0.122 | 0.119 | 0.088 | 0.258 | **0.741** |
| NC | **0.591** | 0.242 | **0.458** | 0.022 | **0.483** | **0.479** | 0.183 | **0.389** | **0.394** | 0.083 | 0.138 | 0.122 | 0.094 | 0.252 | **0.743** |
| GD | **0.529** | -0.227 | **0.783** | **0.709** | **0.796** | **0.914** | **0.491** | -0.059 | 0.250 | 0.256 | **0.581** | **0.398** | 0.308 | **0.486** | **0.440** |
| GC | **0.600** | **-0.590** | -0.034 | **0.654** | **0.388** | 0.260 | **0.856** | -0.214 | -0.047 | -0.118 | 0.319 | 0.023 | -0.058 | 0.158 | 0.235 |
| EV | **0.684** | **-0.399** | **0.490** | **0.766** | **0.678** | **0.683** | **0.691** | -0.045 | 0.091 | 0.117 | **0.518** | 0.268 | 0.179 | **0.355** | **0.428** |
| IV | **0.692** | **-0.430** | **0.423** | **0.754** | **0.650** | **0.650** | **0.799** | 0.028 | 0.146 | 0.099 | **0.471** | 0.248 | 0.190 | **0.389** | **0.386** |
| FE | **0.676** | **-0.385** | **0.377** | **0.662** | **0.413** | **0.522** | **0.629** | 0.276 | 0.306 | **0.357** | **0.463** | **0.397** | 0.288 | **0.464** | **0.496** |
| IT | **0.670** | -0.321 | **0.421** | **0.573** | **0.417** | **0.553** | **0.579** | 0.350 | **0.366** | **0.421** | **0.458** | **0.445** | 0.321 | **0.490** | **0.537** |
| PT | 0.253 | 0.010 | **0.926** | **0.436** | **0.666** | **0.894** | 0.112 | 0.072 | **0.452** | **0.397** | **0.510** | **0.497** | **0.531** | **0.532** | **0.468** |
| FT | **0.397** | -0.158 | **0.763** | **0.539** | **0.729** | **0.833** | 0.343 | -0.202 | 0.180 | 0.093 | 0.334 | 0.206 | 0.166 | 0.246 | **0.469** |
| BF | **0.694** | -0.350 | **0.375** | **0.613** | **0.565** | **0.546** | **0.678** | 0.179 | 0.211 | 0.133 | **0.417** | 0.229 | 0.160 | **0.361** | **0.572** |
| VF | **0.643** | **-0.430** | **0.384** | 0.722 | **0.577** | **0.565** | **0.695** | 0.079 | 0.132 | 0.162 | **0.499** | 0.290 | 0.175 | **0.376** | **0.432** |
| SS | 0.261 | -0.166 | **0.471** | **0.560** | **0.597** | **0.585** | **0.471** | -0.140 | -0.020 | -0.057 | 0.268 | 0.044 | 0.057 | 0.087 | -0.036 |
| FS | 0.335 | -0.211 | **0.492** | **0.626** | **0.586** | **0.611** | **0.502** | -0.031 | 0.016 | 0.064 | 0.335 | 0.155 | 0.136 | 0.191 | 0.016 |
| ES | 0.334 | -0.195 | **0.498** | **0.617** | **0.590** | **0.619** | **0.498** | -0.026 | 0.037 | 0.065 | 0.337 | 0.153 | 0.145 | 0.197 | 0.036 |

**Table S2.** (continued)

| Variable ^a^ | WP | LP | NP | NC | GD | GC | EV | IV | FE | IT | PT | FT | BF | VF | SS | FS |
| --- | --- | --- | --- | --- | --- | --- | --- | --- | --- | --- | --- | --- | --- | --- | --- | --- |
| AR |  |  |  |  |  |  |  |  |  |  |  |  |  |  |  |  |
| PO |  |  |  |  |  |  |  |  |  |  |  |  |  |  |  |  |
| PD |  |  |  |  |  |  |  |  |  |  |  |  |  |  |  |  |
| NA |  |  |  |  |  |  |  |  |  |  |  |  |  |  |  |  |
| UP |  |  |  |  |  |  |  |  |  |  |  |  |  |  |  |  |
| UR |  |  |  |  |  |  |  |  |  |  |  |  |  |  |  |  |
| EN |  |  |  |  |  |  |  |  |  |  |  |  |  |  |  |  |
| FC |  |  |  |  |  |  |  |  |  |  |  |  |  |  |  |  |
| JA |  |  |  |  |  |  |  |  |  |  |  |  |  |  |  |  |
| JU |  |  |  |  |  |  |  |  |  |  |  |  |  |  |  |  |
| AT |  |  |  |  |  |  |  |  |  |  |  |  |  |  |  |  |
| RH |  |  |  |  |  |  |  |  |  |  |  |  |  |  |  |  |
| AN |  |  |  |  |  |  |  |  |  |  |  |  |  |  |  |  |
| AP |  |  |  |  |  |  |  |  |  |  |  |  |  |  |  |  |
| WP |  |  |  |  |  |  |  |  |  |  |  |  |  |  |  |  |
| LP | 0.270 |  |  |  |  |  |  |  |  |  |  |  |  |  |  |  |
| NP | **0.916** | **0.538** |  |  |  |  |  |  |  |  |  |  |  |  |  |  |
| NC | **0.881** | **0.520** | **0.957** |  |  |  |  |  |  |  |  |  |  |  |  |  |
| GD | **0.482** | -0.108 | **0.371** | **0.412** |  |  |  |  |  |  |  |  |  |  |  |  |
| GC | **0.418** | -0.076 | 0.300 | 0.194 | **0.535** |  |  |  |  |  |  |  |  |  |  |  |
| EV | **0.522** | -0.176 | **0.369** | **0.383** | **0.855** | **0.774** |  |  |  |  |  |  |  |  |  |  |
| IV | **0.577** | -0.008 | **0.484** | **0.445** | **0.806** | **0.806** | **0.903** |  |  |  |  |  |  |  |  |  |
| FE | **0.499** | 0.028 | **0.397** | **0.389** | **0.699** | **0.680** | **0.790** | **0.777** |  |  |  |  |  |  |  |  |
| IT | **0.515** | 0.088 | **0.425** | **0.428** | **0.694** | **0.579** | **0.725** | **0.736** | **0.961** |  |  |  |  |  |  |  |
| PT | **0.408** | -0.133 | 0.310 | **0.408** | **0.789** | 0.081 | **0.513** | **0.443** | **0.405** | **0.446** |  |  |  |  |  |  |
| FT | **0.445** | 0.005 | **0.367** | **0.418** | **0.831** | **0.392** | **0.717** | **0.586** | **0.557** | **0.560** | **0.760** |  |  |  |  |  |
| BF | **0.692** | 0.124 | **0.610** | **0.594** | **0.703** | **0.748** | **0.834** | **0.882** | **0.848** | **0.819** | **0.379** | **0.587** |  |  |  |  |
| VF | **0.580** | -0.005 | **0.501** | **0.461** | **0.726** | **0.750** | **0.832** | **0.931** | **0.796** | **0.755** | **0.375** | **0.545** | **0.929** |  |  |  |
| SS | -0.007 | -0.086 | -0.052 | -0.017 | **0.636** | **0.371** | **0.606** | **0.609** | **0.564** | **0.532** | **0.358** | **0.504** | **0.432** | **0.523** |  |  |
| FS | 0.067 | -0.098 | 0.008 | 0.036 | **0.702** | **0.449** | **0.665** | **0.677** | **0.684** | **0.638** | **0.417** | **0.533** | **0.506** | **0.597** | **0.965** |  |
| ES | 0.069 | -0.095 | 0.011 | 0.039 | **0.708** | **0.448** | **0.665** | **0.671** | **0.689** | **0.646** | **0.426** | **0.539** | **0.503** | **0.589** | **0.964** | **0.998** |

^a^ Full name of the variables (see Table 1 in the text for details):

| AN: | Annual precipitation |
| --- | --- |
| AP: | Number of air ports of entry |
| AR: | Area |
| AT: | Mean annual temperature |
| BF: | Batch of Entry-exit inspection and quarantine of freight |
| EN: | Endemism score |
| ES: | Expenditures state-owned research and development institutions above county level in the field of natural sciences and technology |
| EV: | Export value of commodities by place of origin |
| FC: | Forest coverage |
| FDL: | The number of all FDLs in each province |
| FE: | Foreign exchange earnings |
| FS: | Funds of state-owned research and development institutions above county level in the field of natural sciences and technology |
| FT: | Freight traffic |
| GC: | Gross domestic product per capita |
| GD: | Gross domestic product |
| IT: | Number of international tourists |
| IV: | Import value of commodities by place of destination |
| JA: | Mean January temperature |
| JU: | Mean July temperature |
| LP: | Number of land ports of entry |
| NA: | Non-agricultural population |
| NC: | Number of cities with ports of entry |
| NP: | Number of ports of entry |
| PD: | Population density |
| PO: | Population |
| PT: | Passenger traffic. |
| RH: | Mean annual relative humidity. |
| SS: | Staffs of state-owned research and development institutions above county level in the field of natural sciences and technology. |
| UP: | Urban population. |
| UR: | Urbanization rate. |
| VF: | Value of Entry-exit inspection and quarantine of freight. |
| WP: | Number of water ports of entry. |
