# Supplementary material for: Spatial Pattern and Determinants of the First Detection Locations of Invasive Alien Species in Mainland China
Source: PLoS One. 2012 Feb 21;7(2):e31734. doi: 10.1371/journal.pone.0031734 (PMC3283667; doi:10.1371/journal.pone.0031734)
Supplement: Table S3 — Relative importance of explanatory variables and root node competitors in order of improvement for the regression tree in Fig. 2 A . (DOC) [file pone.0031734.s003.doc]

**Table S3.** Relative importance of explanatory variables and root node competitors in order of improvement for the regression tree in Fig. 2 A.

| Order | Competitor ^a^ | Split | Improvement | Importance |
| --- | --- | --- | --- | --- |
| Main | Number of international tourists | 77.795 | 14.287 | 100 |
| 1 | Foreign exchange earnings | 338.20001 | 13.707 | 96.16 |
| 2 | Number of water ports of entry | 4.5 | 11.116 | 13.73 |
| 3 | Gross domestic product per capita | 9.85996 | 9.978 | 65.87 |
| 4 | Value of imported commodities | 89.09441 | 9.821 | 67.49 |
| 5 | Value of EEIQ^b^ freight | 1079.38965 | 9.821 | 62.44 |
| 6 | Export value of commodities | 97.77303 | 9.821 | 65.5 |
| 7 | Batch of EEIQ freight | 75.6912 | 9.665 | 0 |
| 8 | Urbanization rate | 51.24343 | 8.967 | 7.35 |
| 9 | Number of cities with ports of entry | 5.5 | 8.582 | 6.77 |
| 10 | Non-agricultural population | 17.921 | 8.575 | 0 |
| 11 | Number of ports of entry | 6.5 | 7.831 | 6.77 |
| 12 | Funds for scientific research | 98.25011 | 6.581 | 14.44 |
| 13 | Expenditures for scientific research | 93.2939 | 6.581 | 14.44 |
| 14 | Domestic freight traffic | 626.32251 | 6.433 | 0 |
| 15 | Gross domestic product | 3.58363 | 5.904 | 0 |
| 16 | Urban population | 36.917 | 5.779 | 0 |
| 17 | Number of air ports of entry | 4.5 | 5.357 | 0 |
| 18 | Annual precipitation | 1565.90002 | 5.357 | 0 |
| 19 | Mean January temperature | 8.4 | 5.356 | 4.12 |
| 20 | Mean annual temperature | 19.4 | 4.364 | 0 |
| 21 | Forest coverage | 32.76 | 4.215 | 2.42 |
| 22 | Endemism score | 578 | 4.004 | 0 |
| 23 | Population density | 4.44115 | 3.944 | 0 |
| 24 | Staffs for scientific research | 26.9326 | 3.943 | 9.26 |
| 25 | Population | 41.3 | 2.846 | 0 |
| 26 | Mean July temperature | 24.05 | 2.795 | 0 |
| 27 | Mean annual relative humidity | 51.85 | 2.768 | 0 |
| 28 | Domestic passenger traffic | 1233.23853 | 2.585 | 0 |
| 29 | Number of land ports of entry | 6.5 | 1.849 | 0 |
| 30 | Area | 39.36425 | 1.295 | 2.42 |

^a^ See Table 1 in the text for details.

^b^ EEIQ: Entry-Exit Inspection and Quarantine.
